# Quality of anesthetist communication with surgical patients in the perioperative setting: a survey at an academic tertiary referral hospital in Ethiopia

**DOI:** 10.1186/s13037-023-00361-0

**Published:** 2023-05-19

**Authors:** Yophtahe Woldegerima Berhe, Temesgen Agegnehu, Mulualem Endeshaw, Nurhusen Riskey, Getasew Kassaw

**Affiliations:** 1 Department of Anesthesia, University of Gondar, Gondar, Ethiopia; 2 Department of Surgery, Debre Markos University, Debre Markos, Ethiopia; 3 Department of Internal Medicine, University of Gondar, Gondar, Ethiopia

**Keywords:** Perioperative anesthetist communication, Perioperative communication, Quality of communication, Clinical communication, Communication

## Abstract

**Background:**

Effective communication is a fundamental step in providing best medical care and recognized as vital component of clinical anesthesia practice. Poor communication adversely affects patients’ safety and outcome. The objective of this study was to investigate the quality of anesthetist communication from patients’ perspectives at University of Gondar Comprehensive Specialized Hospital (UoGCSH), Northwest Ethiopia.

**Methodology:**

A descriptive cross-sectional study was conducted on 423 surgical patients from April 1, – May 30, 2021. Perioperative patient-anesthetist communication (PPAC) was measured by using 15-items Communication Assessment Tool graded by 5-points Likert scale. Data collection was executed during postoperative time as the patients were optimally recovered from anesthesia. The collected data were cleaned and descriptive analysis was performed.

**Results:**

A total of 400 (94.6% response rate) patients included and 226 (56.7%) were female. The median (IQR) age was 30 (25 – 40) years. Three-hundreds and sixty-one (90.3%) patients had reported good PPAC and 39 (9.8%) reported poor PPAC. The median (IQR) of PPAC scores was 53.0 (48.0 – 57.0) and range from 27 to 69. Highest mean score was observed for the item “Talked in terms I could understand” (4.3 ± 0.7). Lowest mean scores were observed for the item “Checked to be sure I understood everything” (1.9 ± 0.9). Patients who had underwent emergency surgery, no previous anesthetic exposure, had significant preoperative anxiety, no history of previous hospital admission, and moderate-severe preoperative pain were found to have poor PPAC compared to their counterparts in the proportions of 82.1%, 79.5%, 69.2%, 64.1%, and 59.0% respectively.

**Conclusions:**

There was good PPAC in our hospital from patients’ perspective. However, there should be improvements in checking the degree of understanding of the delivered information, encouraging to question, disclosing next steps and involving in decision-making. Patients who underwent emergency surgery, had no previous anesthetic exposure, had clinically significant level of preoperative anxiety, had no history of previous hospital admission, and had moderate-severe preoperative pain were found to have poor PPAC.

## Introduction

Effective communication is a fundamental step in providing best medical care and recognized as vital component of clinical anesthesia practice [1, 2]. Communication practices in healthcare have been strongly implicated in the outcomes of surgical patients [3]. Caregivers must provide accurate communication of the important findings, and individualized risk stratification at it benefits both the practitioners and the patients [4].

Poor communication hinders accurate preoperative assessment, which can compromise safety during perioperative management. It was indicated as a primary cause for medical errors and associated mortality and mortality [5, 6]. In-contrast, effective communication facilitates better understanding and potentially improves postoperative outcomes [1, 7]. Medical errors one of the leading causes of mortality and communication failures were indicated as one of the root causes [8–10]. These failures could occur either in patient-caregiver communication or/and inter-disciplinary communication which are the primary dimensions of communication in clinical practice.

A number of barriers and challenges have been identified for communication in healthcare [1, 5, 11–18]. Therefore; variety of approaches and models of communication have been proposed and implemented to facilitate optimal communication and interactions between patients and health professionals [2, 3, 5, 7, 15, 17, 19–24]. However, communication problems have been persisting challenges of clinical practice and multiple barriers has been identified [17, 24]. Hence, scholars urge to provide curriculum-based communication skill training during preservice training of medical programs and many schools have started teaching communication skills. Moreover, training in communication is also now objectively evaluated as a core competency in various accreditation settings in the developed world [17, 25].

The anesthetist usually faces communication challenges that are unique to the circumstances of anesthesia practice. As a key member of the perioperative team, they are expected to develop strong communication and inter-personal skills to enhance interactions with patients and other professionals in the complex inter-disciplinary environment [20, 22, 26]. Communication is not all language. Manners, habits, appearances, and inter-personal skills affect the impression the anesthetist makes on patients. Thus, clear, concise, respectful communication is essential in anesthesia practice [22]. An anesthetist who developed effective inter-personal and communication skills can prevent medical disasters, expensive interventions, and warrant the provision of optimal patient care [10].

Despite the irreplaceable role of communication in healthcare, there is extremely limited research-based evidence regarding patient-caregiver communication in the low- and middle-income parts of the world; particularly, perioperative patient-anesthetist communication (PPAC). Therefore; the objective of this study was to investigate the quality of anesthetist communication with surgical patients in the perioperative setting from patients’ perspectives at University of Gondar Comprehensive Specialized Hospital (UoGCSH), Northwest Ethiopia.

### Methodology

A descriptive cross-sectional study was conducted at University of Gondar Comprehensive Specialized Hospital (UoGCSH) from April 1, – May 30, 2021. The university hospital is the most senior of the four comprehensive specialized hospitals in the Northwest Ethiopia and found in Gondar town. During the data collection time, the hospital had 4 general, 2 obstetric, and 2 gynecologic operation theatres. As documented on the surgical registrations, all operation theatres usually served 20 – 30 surgical patients per day. However, due to COVID-19 pandemic, there was relative decrement in the flow of surgical patients.

All consecutive adult (18 +) patients who underwent surgical operations during the study period were eligible to be included in the study unless they were unwilling to participate, or unable to communicate due to variety of reasons such as head injury and psychiatric illness.

The dependent variable was perioperative patient-anesthetist communication (PPAC) which was measured by using 15-items Communication Assessment Tool (CAT). The tool was developed and declared as a valid and reliable tool to assess patient-physician communication by Makoul et al. It has a 5-points Likert scale which graded each item as Poor [1], Fair [2], Good [3], Very Good [4], and Excellent [5]. The CAT score ranges from 5 to 75 and PPAC was considered as adequate communication when CAT score was greater than or equal to 45 and below 45 was considered as inadequate communication [27].

The independent variables were socioeconomic-demographic factors (sex, age, educational status, occupational status, residency, and marital status), behavioral factors (alcoholism, smoking, and substance abuse), and clinical factors (ASA physical status, preoperative anxiety, previous anesthetic exposure, type, urgency, and grade of the current surgical operation).

Sample size was determined by using single population proportion formula was used at 50% proportion; 95% of confidence interval and 5% margin of error. The calculated sample size was 384; and when 10% of non-response rate was added, the total sample size became 423. All consecutive and eligible surgical patients were included in the study.

Ethical approval was obtained from Ethical Review Committee of Department of Anesthesia, University of Gondar (Reference number: ANST13/02/2021). Informed consent was obtained from each study participants after brief explanation about the study. Confidentiality was ensured by removing identifiers. All methods were performed in accordance with the relevant guidelines and regulations.

Data collection was executed by an anesthetist during postoperative time as the patients were optimally recovered from anesthesia. The collected data were cleaned and analyzed by using SPSS version 20 software (IBM Corporation). Descriptive analysis was performed and presented by using frequency, percentage, mean, median, and ranges. Relationships among variables were presented in cross-tabulations.

## Results

A total of 423 patients were approached for data collection and data from 400 (94.6f%) participants were used for final analysis. Whereas, data from 7 (1.7%) patients were excluded due to incompleteness. The 226 (56.7%) patients were female and majority of the participants 189 (47.3%) had age between 18 and 29. The median (IQR) age was 30 (25 – 40) years. Patients who came from rural residencies count 237 (59.3%) and 168 (42.0%) participants had not attended formal education while only 70 (17.5%) had accomplished college education (Table 1).

**Table 1:** Socio-demographic and clinical characteristics of participants and their relation to patterns of patient-anesthetist communication, *N*: 400

Variables	Classifications	Frequency (n (%))	Quality of anessthetist communication	
			**Adequate (n (%))**	**Inadequate (n (%))**
Sex	Male	174 (43.5)	156 (43.2)	18 (46.2)
	Female	226 (56.3)	205 (56.3)	21 (53.8)
Age	18 – 29	189 (47.3)	171 (47.4)	18 (46.2)
	30 – 39	110 (27.5)	98 (27.1)	12 (30.8)
	40 – 49	52 (13.0)	47 (13.0)	5 (12.8)
	50 – 59	25 (6.3)	23 (6.4)	2 (5.1)
	≥ 60	24 (6.0)	22 (6.1)	2 (5.1)
Residence	Urban	163 (40.8)	146 (40.4)	17 (43.6)
	Rural	237 (59.3)	215 (59.6)	22 (56.4)
Language (Mother-tongue)	Amharic	386 (96.6)	359 (99.4)	27 (69.2)
	Others	14 (3.4)	2 (0.6)	12 (30.8)
Educational status	Illiterate	168 (42.0)	148 (41.0)	20 (51.3)
	Primary school	83 (21.0)	78 (21.6)	5 (12.8)
	Secondary school	79 (19.8)	70 (19.4)	9 (23.1)
	College and above	70 (17.3)	65 (18.0)	5 (12.8)
Previous hospital admission	Yes	148 (37.0)	134 (37.1)	14 (35.9)
	No	252 (63.0)	227 (62.9)	25 (64.1)
Previous anesthetic exposure	Yes	91 (22.8)	83 (23.0)	8 (20.5)
	No	309 (77.3)	278 (77.0)	31 (79.5)
ASA physical status	Class I	205 (51.3)	189 (52.4)	16 (41.0)
	Class II	195 (48.8)	172 (47.6)	23 (59.0)
Type of surgery	Elective	145 (36.3)	138 (38.2)	7 (17.9)
	Emergency	255 (63.8)	223 (61.8)	32 (82.1)
Class of surgery	General and urologic	124 (31.0)	114 (31.6)	10 (25.6)
	Orthopedic	88 (22.0)	79 (21.9)	9 (23.1)
	Gynecologic	63 (15.8)	59 (16.3)	4 (10.3)
	Obstetric	125 (31.3)	109 (30.2)	16 (41.0)
Type of anesthesia	General anesthesia	165 (41.3)	151 (41.8)	14 (35.9)
	Regional anesthesia	235 (58.8)	210 (58.2)	25 (64.1)
Preoperative pain	No pain	58 (14.5)	54 (15.0)	4 (10.3)
	Mild pain	129 (32.3)	117 (32.4)	12 (30.8)
	Moderate-severe pain	213 (53.3)	190 (52.6)	23 (59.0)
Postoperative pain	No pain	55 (13.8)	50 (13.9)	5 (12.8)
	Mild pain	281 (70.3)	254 (70.4)	27 (69.2)
	Moderate-severe pain	64 (16.0)	57 (15.8)	7 (17.9)
Preoperative anxiety	Yes	237 (59.3)	210 (58.2)	27 (69.2)
	No	163 (40.8)	151 (41.8)	12 (30.8)

*ASA* American society of anesthesiologists

Concerning clinical characteristics of participants, slightly over the half (205 (51.2%)) were classified as ASA-I and the remaining 195 (48.8%) patients as ASA-II. The 148 (37.0%) patients had previous hospital admissions while 91 (22.8%) had previous anesthetic exposure. Nearly 2/3^rd^ patients came for emergency surgery and 213 (53.3%) patients had complained for moderate to severe preoperative pain on visual analogue scale (VAS). The median (IQR) of preoperative pain was 35 (13 – 59) and postoperative pain was 10 (5.0 – 21.8). The primary plan of anesthesia was regional anesthesia for 235 (58.8%) surgical patients (Table 1).

Out of 400 surgical patients, 361 (90.3%) had reported good PPAC and 39 (9.8%) reported poor PPAC. The median (IQR) of PPAC scores was 53.0 (48.0 – 57.0) and range from 27 to 69. The highest mean ± SD scores were observed for items “treated me with respect (4.3 ± 0.6),” “talked in terms I could understand” (4.3 ± 0.7), and “the anesthetist team treated me with respect” (4.3 ± 0.6). Whereas, the lowest mean ± SD score was recorded for an item “checked to be sure I understood everything” (1.9 ± 0.9) (Table 2).

**Table 2:** Communication Assessment Tool (CAT) scores, mean and standard deviations (SD), *N* = 400

**Measurements**	**Communication Assessment Tool Items**														
	Greeted me in a way that made me feel comfortable	Treated me with respect	Showed interest in my ideas about my health	Understood my main health concerns	Paid attention to me	Let me talk without interruptions	Gave me as much information as I wanted	Talked in terms I could understand	Checked to be sure I understood everything	Encouraged me to ask questions	Involved me in decisions as much as I wanted	Discussed next steps	Showed care and concern	Spent the right amount of time with me	The anesthetist team treated me with respect
Mean ± SD	4.2 ± 0.7	4.3 ± 0.6	3.7 ± 0.8	3.8 ± 0.7	3.8 ± 0.7	4.0 ± 0.8	3.3 ± 0.8	4.3 ± 0.7	1.9 ± 0.9	2.1 ± 1.0	3.0 ± 0.9	2.0 ± 0.9	4.1 ± 0.7	3.5 ± 1.1	4.3 ± 0.6

Among 39 patients who had reported poor PPAC, 21 (53.8%) were females, 22 (56.4%) were living in rural residencies, 20 (51.3%) were illiterates, 25 (64.1%) had no history of previous hospital admission, 31 (79.5%) had no previous anesthetic exposure, 32 (82.1%) underwent emergency surgery, 23 (59.0%) had complained for moderate-severe preoperative pain, and 27 (69.2%) were found to have clinically significant level of preoperative anxiety (Table 1).

**Fig. 1:**
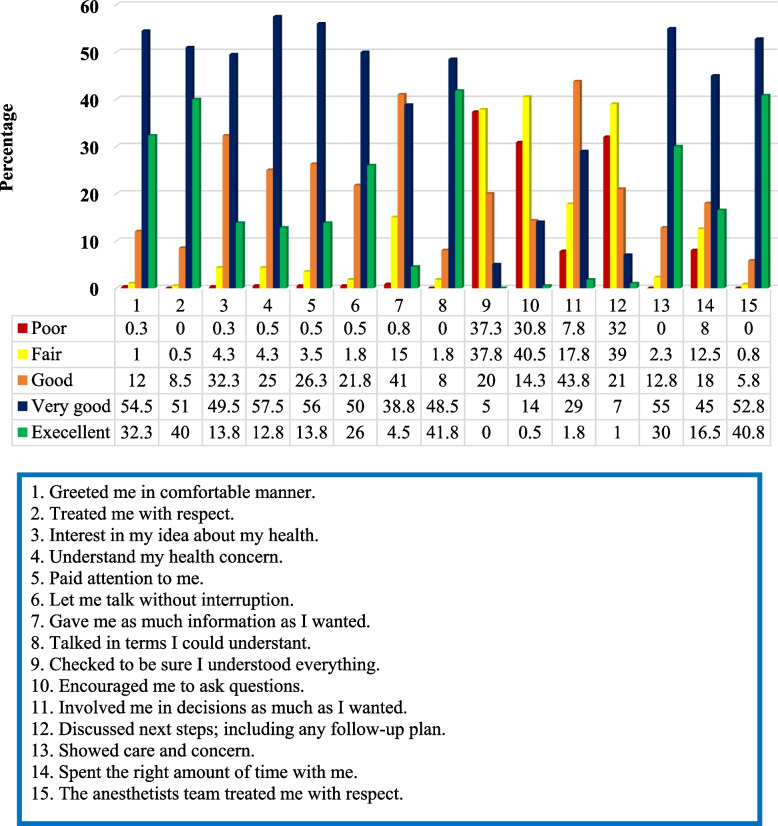
Patients’ perceptions on the quality of perioperative patient-anesthetist communication; *N* = 400

Among the whole study participants (400), 54.5% believed that the anesthetist greeted them in a “very good” manner and 32.3% in “excellent” manner. About 51.0% and 40.0% patients answered “very good” and “excellent” respectively for an item “treated me with respect.” Around 57.5% of participants responded “very good” for an item “understand my health concern.” About 97.8% of the participants acknowledged that the anesthetist “let them talked without interruptions” as they rated “good-to-excellent” for the item. About 99.4% of the patients reported that “the anesthetists team treated them with respect” as they rated “good-to-excellent” for the item (Fig. 1).

Only 4.5% of patients rated “excellent” for an item “give me as much information as I want” while the majority (41.0%) rated it “good.” None of the patients rated “excellent” for an item “checked to be sure I understood everything” while 37.3% and 37.8% of patients rated this item “poor” and “fair” respectively. Large proportion of patients believed that anesthetists did not encourage to ask questions. Only 0.5% of patients had responded for an item “encouraged me to ask questions” while 30.8% and 40.5% responded “poor” and “fair” respectively. Over 2/3^rd^ of the patients responded neither “very good” nor “excellent” for involvement of themselves in decision-making as much as they wanted. Only 1.8% of patient rated “excellent” for their degree of involvement in decision making. Furthermore, nearly 3/4^th^ of the patients (71.0%) responded either “poor” or “fair” for discussion of next steps such as follow-up plans (Fig. 1).

## Discussion

Patient to physician interaction has changed profoundly in the past decades as health policy makers have advocated for shared decision-making and patient-centered care provision. The Ministry of Health of Ethiopia has commenced implementing Careful, Respectful, and Compassionate care (CRC) models of healthcare to enhance patient-centered care. Even though, these models remain still aspirational [24, 28].

In our study, over 90% of the patients believed that there was good PPAC. This high result might be explained by high language homogeneity as 96.6% of patients identified Amharic language as their mother-tongue language which is the official language of the country (Ethiopia). Language is a significant determinant of patient-physician communication. Limited health literacy impedes patient–physician communication, but its effects vary with language concordance. For language discordant individuals, language barriers may surpass limited health literacy in impeding interactive communication [1, 11, 16, 29]. Supporting this, the second highest mean score was observed for the item “talked in terms I could understand” (4.3 ± 0.7).

Higher frequencies of poor PPAC were occurred among those who underwent emergency surgery 32 (82.1%), had no previous anesthetic exposure 31 (79.5%), had clinically significant level of preoperative anxiety 27 (69.2%), had no history of previous hospital admission 25 (64.1%), and had complained for moderate-severe preoperative pain 23 (59.0%) compared to their counterparts. All of the above conditions are high likely to deter the process of effective communication.

The communication process has four main elements (*the sender*, *the message*, *the receiver*, and *the feedback*). Effective communication involves listening, questioning, and assessing the degree of understanding of the transmitted information. Problems in any of these components can result in defective communication [15]. In our study, we found the lowest mean scores for the items “checked to be sure I understood everything” (1.9 ± 0.9), “discussed next steps” 2.0 ± 0.9, and “Encouraged me to ask questions” 2.1 ± 1.0. These showed that there are gaps in assessing the degree of understanding of the transmitted information; and allowing patient to ask questions and know what would follow next. Most of the patients did not believe that they have been adequately involved in decision-making regarding their care. These results are lower compared to a study conducted in the United States of America. The difference might be explained by differences in clinical settings and populations. The former study was done in highly developed settings on patient that were not exclusively surgical [27].

Patient-centered communication has been widely recommended as an essential component of high-quality healthcare and it includes three core values:

- (i) Considering patients’ needs, wants, perspectives and individual experiences,
- (ii) Offering patients opportunities to provide input into and participate in their care and
- (iii) Enhancing partnership and understanding in the patient-physician relationship [30].

This study was one of the few quantitative studies done on PPAC to-date as most of the available studies were qualitative studies. However, the study did not determine the barriers and factors associated with PPAC.

## Conclusions

There was good PPAC in our hospital from patients’ perspective. However, there should be improvements in checking the degree of understanding of the delivered information, encouraging to question, disclosing next steps and involving in decision-making. These may facilitate to achieve the implementation of patient-centered care targeted by Careful, Respectful, and Compassionate care models directed by the Ministry of Health of Ethiopia. Patients who underwent emergency surgery, had no previous anesthetic exposure, had clinically significant level of preoperative anxiety, had no history of previous hospital admission, and had moderate-severe preoperative pain were found to have poor PPAC.

## Data Availability

Data and materials used in this study are available and can be presented by the corresponding author upon reasonable request.

## Abbreviations

ASA: American society of anesthesiologists
CAT: Communication assessment tool
COVID-19: Corona virus disease-2019
IQR: Inter-quartile range
PPAC: Perioperative patient-anesthetist communication
SPSS: Statistical package for the social sciences
UoGCSH: University of Gondar Comprehensive Specialized Hospital
